# Hedgehog signaling activation required for glypican-6-mediated regulation of invasion, migration, and epithelial–mesenchymal transition of gastric cancer cells

**DOI:** 10.1042/BSR20193181

**Published:** 2020-06-15

**Authors:** Chen Zeng, Ran Yan, Guanghua Yang, Sen Xiang, Fuli Zhao

**Affiliations:** 1 Department of Medical Oncology, Central Hospital, Zhumadian City 463000, China; 2 Office of Huanghuai University, Zhumadian City 463000, China

**Keywords:** Gastric cancer, Glypican-6, Hedgehog, Invasion, Migration

## Abstract

Gastric cancer (GC) is the fifth most common cancer worldwide and one of the most aggressive cancers in China. Glypican 6 is highly expressed in gastric adenocarcinoma and may act as a diagnostic and prognostic marker; however, the functional importance and molecular mechanism of glypican 6 in GC remains unclear. In the current study, we aimed to reveal the function and mechanism of glypican 6 in two GC cell lines: MKN-45 and SGC-7901. We found higher expression of glypican 6 in MKN-45 and SGC-7901 cells than in cells from the normal gastric mucosa epithelial cell line GES-1. Glypican 6 knockdown suppressed MKN-45 and SGC-7901 cell proliferation. A Transwell assay confirmed that glypican 6 silencing inhibited the migration and invasiveness of MKN-45 and SGC-7901 cells. Epithelial-to-mesenchymal transition (EMT) markers were determined by western blotting, and the results showed reduced Vimentin expression and elevated E-cadherin expression in glypican 6 short interfering RNA (siRNA) transfected MKN-45 and SGC-7901 cells. However, glypican 6 overexpression in GES-1 cells showed no significant promotion on GES-1 cells proliferation and migration. Further studies confirmed that glypican 6 siRNA regulated Hedgehog and Gli1 signaling and participated in the function of glypican 6 on MKN-45 and SGC-7901 cell migration and invasion. Our findings suggest that decreased glypican 6 expression inhibits the migration and invasion ability of GC cells.

## Introduction

Gastric cancer (GC), one of the most common cancers, is the third-most lethal cancer in the world after liver and lung cancers [1,2]. Approximately 990000 new cases of GC are diagnosed worldwide, and approximately 738000 patients die from this disease annually [3]. Although the incidence of GC has declined recently, it remains the fourth-most common malignancy in the world [2]. Evidence has confirmed that mortality and morbidity associated with this disease are highest in East Asia, including Japan, Mongolia, Korea, and China, and it is the second-leading cause of cancer mortality in China [4]. Despite recent advances in diagnosis, the diagnosis of GC remains a challenge, and most patients are not diagnosed until late stages of the disease [5]. Chemotherapy, surgery, or a combination of therapies can improve patient survival rates, but the median survival time is approximately 12 months for patients with advanced GC [6–8]. It is therefore meaningful to identify effective targets and related mechanisms to develop useful therapeutic strategies for GC [9,10].

The proteoglycan proteins, called glypicans, have glycosylphosphatidylinositol anchors that attach to plasma membranes [11]. Mammals share six common glypicans, 1–6 [12]. Glypican members play important roles in cancer. Glypican 1 is widely expressed in pancreatic cancer, glioma, and breast carcinoma, and low levels of glypican 1 inhibit metastasis, growth, and tumorigenicity [13]. Glypican 6 is a target of nuclear factor of activated T-cells, and it modulates breast cancer cell invasiveness and migration [13]. Dinccelik-Aslana et al. [14] found that glypican 5 and glypican 6 gene expression was significantly higher in GC tumor tissues than in a normal sample. However, the function of glypican 6 remains unclear.

Glypicans can stimulate or inhibit signaling activity, including the Hedgehog (Hh) signaling pathway [15]. The Hh signaling pathway is implicated in tissue patterning, embryonic development, and cell differentiation [16]. Hyperactivation of this signaling pathway is associated with cancer progression in medulloblastoma and basal cell carcinoma [17]. The Gli family of transcription factors affects cell proliferation, migration, and differentiation by regulating cell-type-specific gene expression [18]. Chen et al. showed that Gli1 expression acts as a prognostic biomarker in patients with GC. Positive Gli1 expression is associated with poor-prognosis gross and histological types, advanced TNM tumor classification, and large tumor size [19]. The up-regulation of Gli1 participates in galectin-1 induced epithelial–mesenchymal transition (EMT), migration, and invasiveness of GC cells [20].

In the present study, we investigated the effects of glypican 6 on the GC cell lines MKN-45 and SGC-7901 cells. We confirmed that glypican 6 knockdown suppressed GC cell proliferation, migration, and invasiveness. The Hh and Gli1 signaling pathway was inhibited in MKN-45 and SGC-7901 cells that were transfected with glypican 6 siRNA. These results indicate that the Gli1 signaling pathway plays crucial roles in the function of glypican 6 on MKN-45 and SGC-7901 cells.

## Methods

### Cell culture

Human GC cell lines MKN-45 and SGC-7901, and normal gastric mucosa epithelial cell line GES-1 were obtained from ATCC (Manassas, VA, U.S.A.). Cells were maintained in Dulbecco's Modified Eagle's medium (DMEM) supplemented with 10% fetal bovine serum, streptomycin (100 μg/ml), and penicillin (100 U/ml).

### Cell transfection

MKN-45 and SGC-7901 cells were untreated or treated with purmorphamine (Gli1 agonist, TESTMART, China) or GANT 61 (Gli1/2 inhibitor, abcam, ab120904) and then transfected with glypican 6 siRNA (Tsingke, Beijing, China) and the control scramble siRNA (NC) using Lipofectamine 2000 according to the manufacturer's protocol. Three siRNA oligo for knocking down glypican 6 were identified. The sequence with best effect is: forward 5′-CGG CTG GTC ACA GAC ATA AA-3′ and reverse 5′-CTC GTC CTT GCA GAT AGT GTA G-3′. The siRNA sequence for NC was as follows: forward 5′-CTC CTC CAC CTT TGA CGC TG-3′ and reverse 5′- TCC TCT TGT GCT CTT GCT GG-3′.

### Cell Counting Kit-8 assay

Cell Counting Kit-8 (CCK-8) method was employed to detect cell proliferation. Untreated cells and cells treated with purmorphamine or GANT 61 were transfected with glypican 6 siRNA and the control scramble siRNA for 48 h. Then, 10 μl of CCK-8 solution (5 mg/ml) was added to each well for 4 h incubation. The absorbance at 570 nm was read with a plate reader (Thermo Fisher Scientific, Waltham, MA, U.S.A.).

### Transwell assay

Cell invasion and migration were assayed with a 24-well Transwell membrane coated with or without Matrigel™ (BD Biosciences, San Jose, CA, U.S.A.) according to the method described previously [21]. The MKN-45 and SGC-7901 cells were starved with serum-free DMEM. Then, 5 × 10^4^ cells in serum-free DMEM were seeded in the upper chamber and DMEM containing 10% FBS was added to the lower chamber as the chemoattractant. Forty-eight hours later, non-migrating and non-invading cells on the upper surface of the membrane were removed, while migrated or invaded cells on the lower surface of the membrane were fixed with 4% paraformaldehyde and stained with 0.5% crystal violet.

### Quantitative Real-time PCR (qRT-PCR)

Total RNA from MKN-45 and SGC-7901 cell samples was obtained using TRIzol reagent (Invitrogen Life Technologies, Carlsbad, CA, U.S.A.) according to the protocol of the manufacturer. A TransScript First-strand cDNA Synthesis SuperMix kit was used to cDNA synthesis. Quantitative RT-PCR was performed using a SYBR Premix Ex Taq kit (TaKaRa, Japan). Targeted gene expression was calculated using the 2^−ΔΔCt^ method while β-actin was used as internal control.

### Western blot

MKN-45 and SGC-7901 cells were lysed using RIPA buffer (Sigma–Aldrich, Germany), and the protein concentration was determined by BCA kits. 20 μg of total protein samples were separated by SDS-PAGE. Then proteins were transferred to a polyvinylidene fluoride (PVDF) membrane. The membranes were blocked with 5% non-fat dry milk and incubated with corresponding primary antibodies (Abcam, Cambridge, MA, U.S.A.) overnight at 4 °C, followed by incubation with secondary antibodies for 1 h at room temperature. The primary antibodies were as follows: rabbit polyclonal to glypican 6 antibody (1:100), rabbit polyclonal to Gli1 antibody (1:1000), rabbit polyclonal to E-cadherin antibody (1:500), mouse monoclonal to Vimentin antibody (1:500), and rabbit polyclonal to N-cadherin antibody (1 µg/ml). The second antibodies were Horseradish-peroxidase-rabbit anti-mouse IgG and horseradish-peroxidase-goat anti-rabbit IgG. The reactive bands were visualized using enhanced chemiluminescence reagents (Thermo-Pierce, Rockford, IL, U.S.A.), and the gray values of each band were calculated using Image J software. The gray values of targeted protein were normalized to β-actin, and the different treatment groups were normalized to control group.

### Statistical analysis

The statistical analyses were performed using SPSS version 19.0 (SPSS, Inc., Chicago, IL). The data are expressed as mean ± SD. Significance analyses were performed using one-way analysis of variance (ANOVA), followed by post hoc Bonferroni's *t* tests. Statistical significance was set at *P*<0.05.

## Results

### Glypican 6 was highly expressed in gastric cancer cells

**Figure 1:**
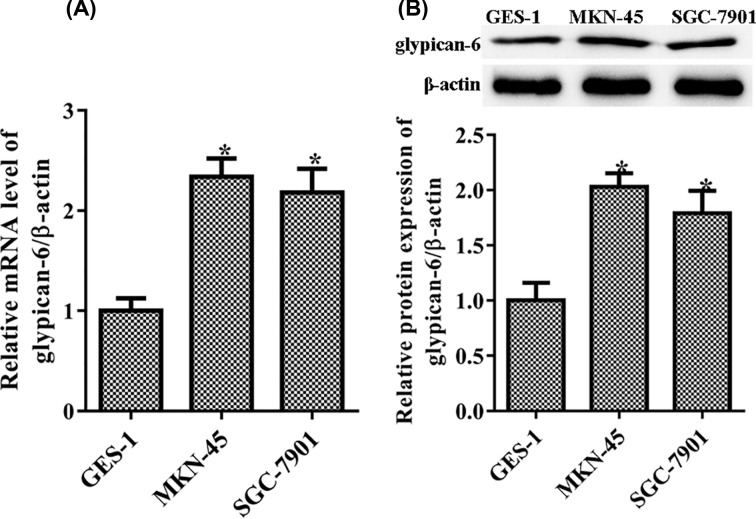
Glypican 6 increased in MKN-45 and SGC-7901 cells (**A**) Glypican 6 mRNA was measured by RT-qPCR. (**B**) Glypican 6 protein expression was measured by western blot. Data are expressed as mean ± SD. *P<0.05 versus the GES-1 cells.

The level of glypican 6 in GC cell lines MKN-45 and SGC-7901 was compared with that of normal gastric mucosa epithelial cell line GES-1. The mRNA for glypican 6 was measured via RT-qPCR, and higher levels in the MKN-45 (*P* = 0.031) and SGC-7901 cells (*P* = 0.036) than in the GES-1 cells were observed (Figure 1A). Consistent with RT-qPCR results, glypican 6 protein levels by western blot analyses were much higher in MKN-45 cells (*P* = 0.026) and SGC-7901 cells (*P* = 0.04) than in GES-1 cells (Figure 1B).

### Suppressing glypican 6 expression regulated cell proliferation

**Figure 2:**
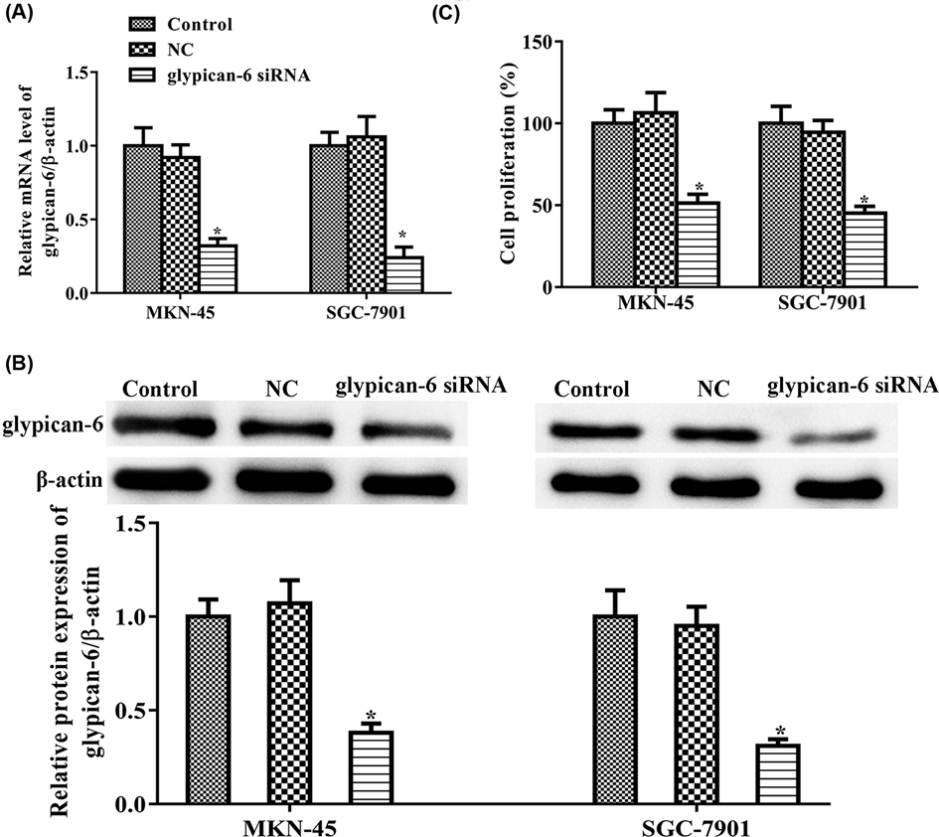
Glypican 6 silencing inhibited MKN-45 and SGC-7901 cell proliferation (**A**) Glypican 6 mRNA was measured by RT-qPCR. (**B**) Glypican 6 protein expression was measured by western blot. (**C**) Cell proliferation was detected by CCK-8 assay. Data are expressed as mean ± SD. * *P*<0.05 versus the control.

To further reveal the effects of glypican 6 on MKN-45 and SGC-7901 cell biology, glypican-6-silenced MKN-45 and SGC-7901 cells were built. As shown in Figure 2A,B, MKN-45 and SGC-7901 cells transfected with glypican 6 siRNA exhibited very low mRNA and protein expression of glypican 6, compared with cells in the control and scramble siRNA (NC) groups. We next detected the role of glypican 6 deletion in cell proliferation, and the results indicated that glypican 6 silencing markedly suppressed cell proliferation of MKN-45 (*P* = 0.003) and SGC-7901 cells (*P* = 0.006) when compared with the control group (Figure 2C), whereas no significant change was observed between the NC and control groups (Figure 2C, MKN-45: *P* = 0.09; SGC-7901: *P* = 0.09).

### Suppressing glypican 6 expression regulated cell invasion, migration, and epithelial–mesenchymal transition

**Figure 3:**
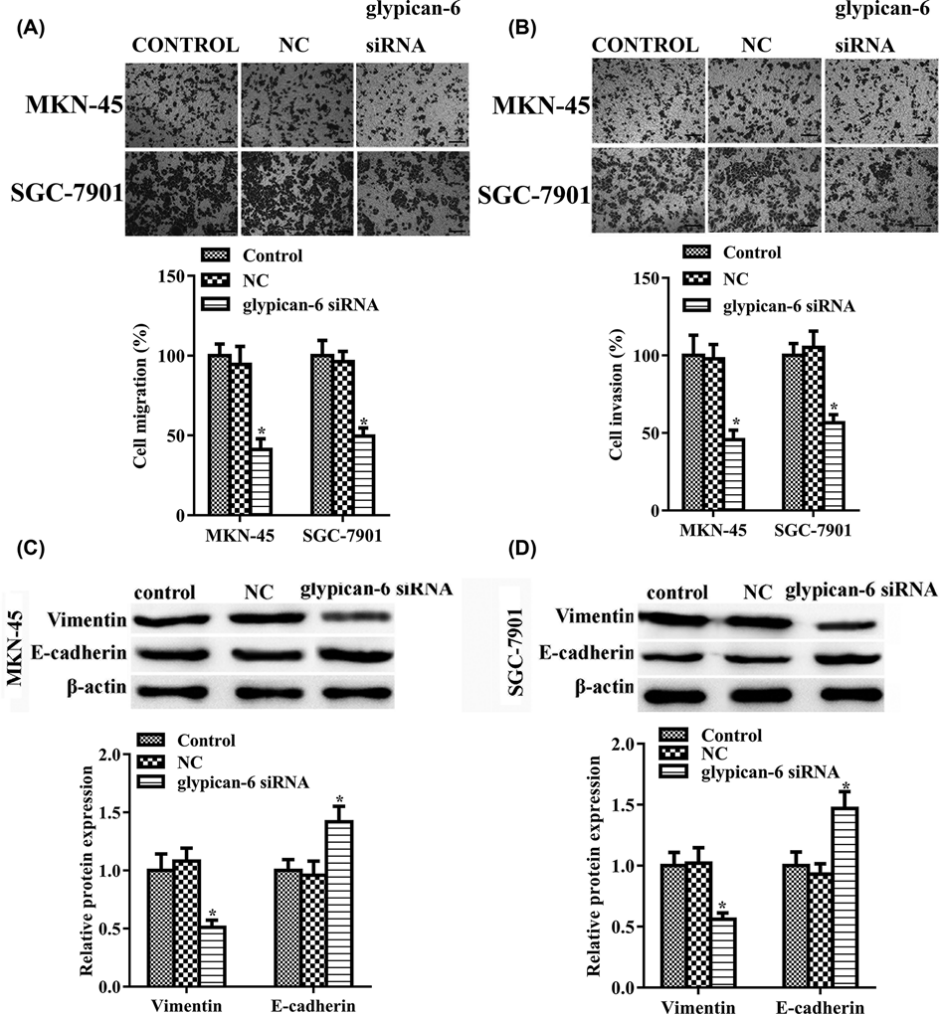
Glypican 6 silencing suppressed cell migration, invasiveness, and EMT in MKN-45 and SGC-7901 cells (**A**) Cell migration and (**B**) invasion were measured by Transwell assay. (**C,D**) E-cadherin and vimentin protein expression were determined by western blot assay in MKN-45 and SGC-7901 cells. Data are expressed as mean ± SD. * *P*<0.05 versus the control. Scale bar = 100 μm.

Cell migration and invasion exacerbates cancer metastasis and progression [22]. Transwell assay result showed that knockdown of glypican 6 markedly suppressed the migration (MKN-45: *P* = 0.021; SGC-7901: *P* = 0.044) and invasiveness (MKN-45: *P* = 0.016; SGC-7901: *P* = 0.037) of both cell lines, compared with cells in the control group (Figure 3A,B). NC transfection had no similar effect (Figure 3A,B, MKN-45: *P* = 0.09; SGC-7901: *P* = 0.10). We next evaluated the effects of glypican 6 depletion on the expression of E-cadherin and Vimentin, two EMT markers. As shown in Figure 3C,D, compared with the control group, glypican 6 knockdown increased E-cadherin expression, whereas it decreased vimentin expression in MKN-45 cells and SGC-7901 cells.

**Figure 4:**
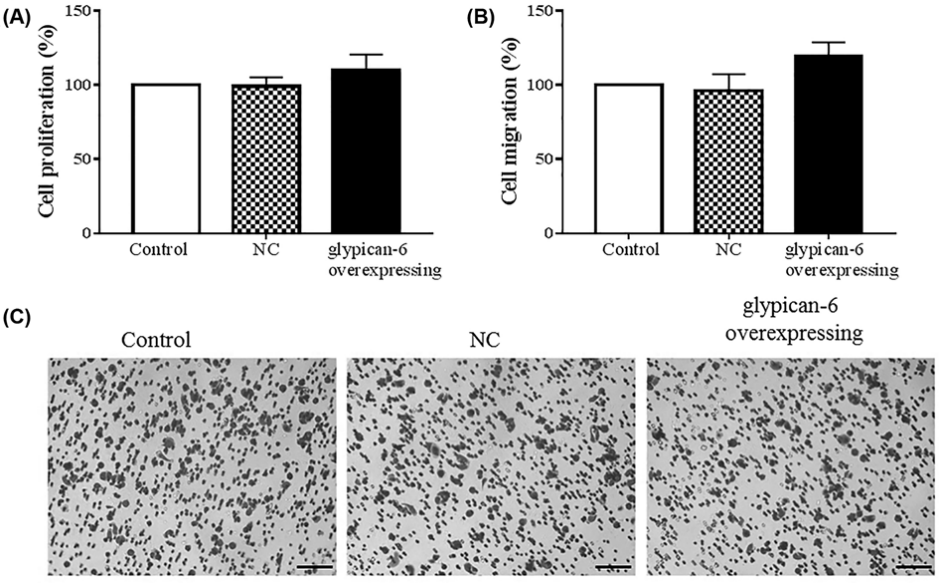
Glypican 6 overexpression showed no significant effect on GES-1 cells proliferation and migration Cell proliferation was detected by CCK-8 assay (**A**) and cell migration was measured by Transwell assay (**B,C**). Data are expressed as mean ± SD.

Besides, we also constructed glypican 6 overexpressed GES-1 cells, and found that overexpression of glypican 6 did not have obvious effect on GES-1 cells proliferation (Figure 4A, *P* = 0.1) and cell migration (Figure 4B,C, *P* = 0.07).

### Gli1 expression is decreased in glypican 6 silencing cells

**Figure 5:**
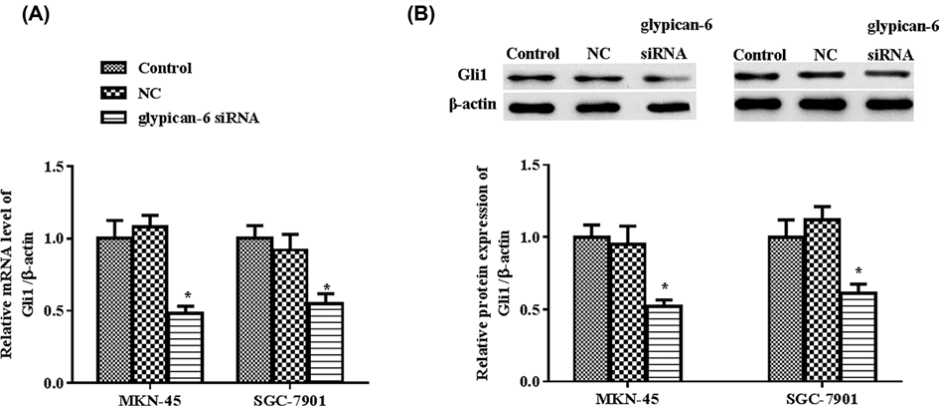
Glypican 6 silencing inhibited the expression of Gli1 in MKN-45 cells and SGC-7901 cells (**A**) Glypican 6 mRNA was measured by RT-qPCR. (**B**) Glypican 6 protein expression was measured by western blot. Data are expressed as mean ± SD. * *P*<0.05 versus the control.

The Hh signaling pathway is implicated in GC [23]. The expression of Gli1 mRNA and protein was measured by RT-PCR and western blot assay. Compared with control group, glypican 6 knockdown significantly decreased Gli1 expression in MKN-45 (*P* = 0.016) and SGC-7901 cells (*P* = 0.022) at the mRNA levels (Figure 5A). The protein level of Gli1 showed similar trend (MKN-45: *P* = 0.009; SGC-7901: *P* = 0.011). No significant difference was observed between the control and NC groups both in MKN-45 and SGC-7901 cells (Figure 5B).

### Hedgehog and Gli1 signaling participated in glypican-6-induced proliferation, invasion, migration, and epithelial–mesenchymal transition

**Figure 6:**
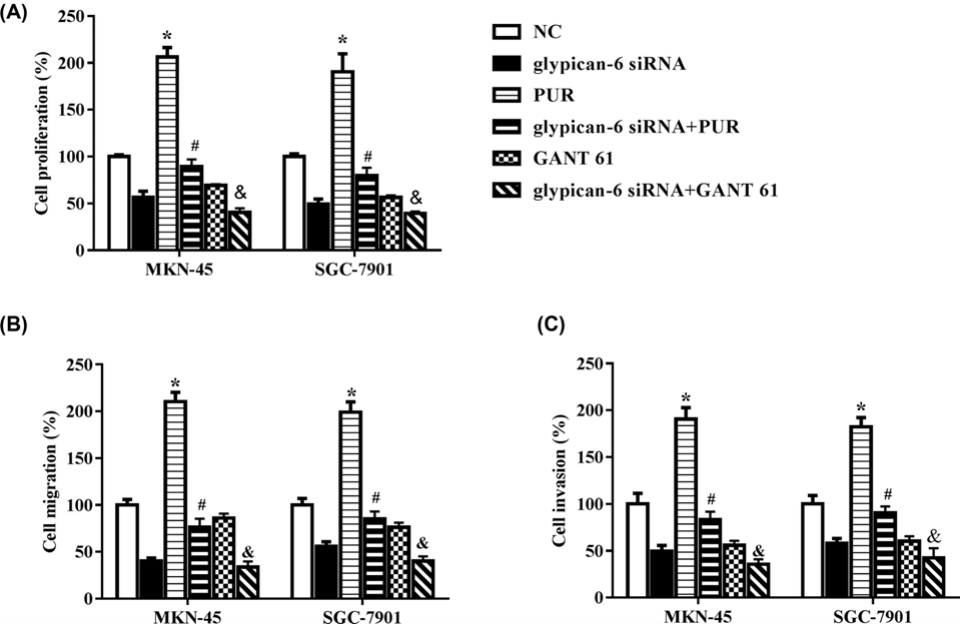
Gli1 activation reversed the effects of glypican 6 silence on MKN-45 and SGC-7901 cell biology (**A**) Cell proliferation was detected by CCK-8 assay. (**B**) Cell migration and (**C**) invasion were measured by Transwell assay. Data are expressed as mean ± SD. * *P*<0.05 versus the NC group and glypican 6 siRNA group, # *P*<0.05 versus the PUR group, & *P*<0.05 versus the GANT 61 group.

To address whether Hh/Gli1 signaling participated in glypican 6 silencing induced cell proliferation, migration, and invasiveness change, MKN-45 and SGC-7901 cells were treated with a Gli1 agonist, purmorphamine or a selective Gli1/2 inhibitor, GANT 61. As shown in Figure 6A, purmorphamine treatment alone markedly increased MKN-45 (*P* = 0.0004) and SGC-7901 cell (*P* = 0.001) proliferation, while GANT 61 treatment significantly decreased cell proliferation (*P* = 0.009 for MKN-45 and *P* = 0.013 for SGC-7901), compared with cells in control group. Purmorphamine co-treatment partially reversed the effects of glypican 6 siRNA on MKN-45 and SGC-7901 cell proliferation (*P* = 0.039 for MKN-45; *P* = 0.044 for SGC-7901), while GANT 61 co-treatment significantly aggravated the reduction effects of glypican 6 siRNA on MKN-45 (*P* = 0.013) and SGC-7901 (*P* = 0.034) cell proliferation. Cell migration and invasion showed similar tendency (Figure 6B,C). These results indicate that Hh/Gli1 signaling plays critical roles in MKN-45 and SGC-7901 cell proliferation and migration. Gli1 signaling may act downstream of the glypican 6 signaling pathway.

## Discussion

As one of the most common cancers worldwide and the second cancer-related death leading cause in China [4], GC remains a significant health problem, with evidence indicating that genetic alterations contribute to GC progression and development [24–26]. The function and mechanism of glypican 6 on GC cells remains unclear. The main aim in this study thus was to reveal the effects of glypican 6 on proliferation, migration, invasiveness, and EMT in cells from the GC cell lines MKN-45 and SGC-7901.

The glypican family has six members, glypicans 1–6. The expression of glypican 3 increases in the atypical multidrug-resistant GC cell line EPG85-257RNOV [27]. The mRNA expression of glypican 6 is higher in GC tumor tissues than in normal tissue [14]. Consistent with these reports, we confirmed that glypican 6 was highly expressed in MKN-45 and SGC-7901 cells.

Glypican proteins participate in the development and progression of liver [28], pancreatic [29], breast [30], and gastric [31,32] cancers. Glypican 5 acts as an oncogene in GC that regulates GC cell proliferation and invasion [33]. Glypican 3 protect the atypical multidrug-resistant GC cell line EPG85-257RNOV against mitoxantrone [27]. Specific gene silencing by siRNA transfection is widely used to study gene function in disease. We studied the effects of glypican 6 silencing on cell biology and found that glypican 6 silence affected proliferation, migration, and invasiveness of MKN-45 and SGC-7901 cells. However, the effect of overexpressed glypican 6 on GES-1 cells was not obvious. The high expression of glypican 6 in GC cells might play important roles in GC development, however, it could not initiate tumorigenesis.

Accumulating evidence suggests that glypicans are associated with multiple morphogens, growth factors, and signaling pathways, such as bone morphogenetic proteins [34,35], fibroblast growth factors [36,37], Wnts [38,39], and Hhs [40–42]. Glypican 5 promotes rhabdomyosarcoma cell proliferation by activating Hh signaling [43]. Glypican 6 activates Hh signaling and thus promotes long-bone growth [11]. Consistent with these reports, our study suggests that glypican 6 inhibition markedly inhibited Gli1 expression both in MKN-45 and SGC-7901 cells. The Hh and Gli1 signaling pathway plays important roles in GC [19]. Gli1 up-regulation promotes GC cell migration, invasiveness, and EMT [20]. Our study demonstrated that Hh and Gli1 signaling affects the function of glypican 6 silencing in MKN-45 and SGC-7901 cell biology.

In conclusion, our study demonstrated that suppressing glypican 6 expression inhibited MKN-45 and SGC-7901 cell proliferation, migration, and invasiveness by inhibiting Hh and Gli1 activation. However, the function of glypican 6 on GC development and progression remains unclear. Further studies in animal or patients are needed.

## Abbreviations

CCK-8: Cell Counting Kit-8
DMEM: Dulbecco's Modified Eagle's medium
EMT: epithelial-to-mesenchymal transition
GC: gastric cancer
Hh: Hedgehog
NC: control scramble siRNA
PVDF: polyvinylidene fluoride
siRNA: short interfering RNA
